# A Multimarker Approach for Early Prediction of Preeclampsia Using Uterine Artery Doppler Pulsatility Index, Hemodynamic, and Biochemical Parameters: A Prospective Observational Study

**DOI:** 10.7759/cureus.110879

**Published:** 2026-06-15

**Authors:** Rhiya Singh, Neena Gupta, Divya Dwivedi

**Affiliations:** 1 Obstetrics and Gynaecology, Ganesh Shankar Vidyarthi Memorial Medical College, Kanpur, IND

**Keywords:** first trimester, mean arterial pressure, mean platelet volume, preeclampsia, pregnancy-associated plasma protein-a, uterine artery doppler

## Abstract

Background

Early identification of preeclampsia remains challenging, particularly in resource-limited settings. Although several clinical, biochemical, and Doppler markers have been explored, their individual predictive performance remains limited, necessitating evaluation of combined approaches.

Objectives

This study assesses the predictive value of mean arterial pressure (MAP), uterine artery Doppler pulsatility index (PI), pregnancy-associated plasma protein-A (PAPP-A), and mean platelet volume (MPV), individually and in combination, for early prediction of preeclampsia.

Materials and methods

In this prospective study, 150 singleton pregnancies were evaluated between 11 and 14 weeks of gestation. MAP, uterine artery Doppler PI, serum PAPP-A, and MPV were measured at recruitment. Participants were followed until delivery. Univariate analysis, logistic regression, receiver operating characteristic analysis, and detection rate assessment were performed. The study was approved by the Institutional Ethics Committee and conducted in accordance with the Declaration of Helsinki.

Results

Preeclampsia was observed in 19 women (12.7%). Among the individual markers, the highest discriminative ability was observed for MAP (AUC 0.84), and the MPV was the only predictor to show significance on multivariate analysis. PAPP-A and uterine artery Doppler PI were significantly different between the normotensive and preeclampsia groups in univariate analysis but did not demonstrate high predictive ability. The four-parameter model incorporating MAP, MPV, PAPP-A, and uterine artery Doppler PI demonstrated the highest predictive ability for preeclampsia (AUC 0.87; sensitivity 83.3%, specificity 81.5%).

Conclusion

The combination of MAP, MPV, PAPP-A, and uterine artery Doppler PI demonstrated higher predictive performance than individual markers in this study population and may support first-trimester risk stratification for preeclampsia.

## Introduction

Preeclampsia is a multisystem hypertensive disorder of pregnancy characterized by the onset of hypertension after 20 weeks of gestation in the presence of proteinuria with or without evidence of maternal or uteroplacental dysfunction [1]. Despite progress in the field of obstetric care, preeclampsia is a major cause of maternal and perinatal morbidity and mortality worldwide [2]. The worldwide occurrence of preeclampsia is estimated to be two to eight percentage of pregnancies, with an inordinately greater burden in low and middle-income countries [3]. In India, the reported prevalence is between 8 and 10%, leading to a massive public health issue given high birth rates and sporadic access to quality antenatal care [4]. Maternal complications include eclampsia, HELLP syndrome, stroke, and long-term cardiovascular disease. Fetal morbidity is primarily caused by placental insufficiency with resulting growth restriction of the fetus and preterm birth [5].

The pathogenesis of preeclampsia is complex and multifactorial, and abnormal placentation is known to be a central initiating event [6]. Inadequate trophoblastic invasion and failure of spiral artery remodeling increase uteroplacental vascular resistance and placental ischemia, which precedes the clinical manifestations of disease [7]. Uterine artery Doppler velocimetry allows the non-invasive assessment of uteroplacental blood flow, and high levels of pulsatility indices (PIs) in early pregnancy are associated with a higher risk of preeclampsia [8].

Biochemical and hematological markers of the early onset of placental and endothelial dysfunction have therefore been investigated for first-trimester risk prediction [9]. Pregnancy-associated plasma protein-A (PAPP-A), a placentally derived biomarker, has been associated with hypertensive disorders of pregnancy, with several studies reporting reduced levels in cases of impaired placentation, although findings may vary depending on the method of expression and population studied [8]. The mean platelet volume (MPV), a marker of platelet activation, is a marker of endothelial injury and systemic inflammation involved in the development of the disease [6]. Mean arterial pressure (MAP), a composite indicator of systolic and diastolic blood pressure, reflects early maternal vascular maladaptation and has been shown to outperform individual blood pressure components in first-trimester prediction of preeclampsia [10]. Although the predictive value of separate markers is restricted, early risk stratification may be improved by the combined evaluation of biophysical, biochemical, and hematological parameters [9]. Early first-trimester screening enables identification of women at high risk of preeclampsia before the onset of clinical symptoms. This facilitates consideration of preventive measures such as low-dose aspirin initiated before 16 weeks of gestation, which has been shown to reduce disease incidence [11].

According to existing evidence, it was hypothesized that alterations in uterine artery Doppler parameters, MAP, MPV, and PAPP-A levels in early pregnancy are associated with the subsequent development of preeclampsia [8-10].

MAP, uterine artery Doppler PI, PAPP-A, and MPV reflect different biological processes involved in the development of preeclampsia, including maternal hemodynamic changes, placental dysfunction, and platelet activation. Therefore, combining these markers may provide a more comprehensive assessment of risk than evaluating each parameter individually. Such an approach may be particularly useful in settings where access to more complex screening algorithms is limited.

The present study aimed to evaluate the predictive performance of uterine artery Doppler indices, PAPP-A, MAP, and MPV, individually and in combination, for the early prediction of preeclampsia.

## Materials and methods

This prospective observational study was conducted in the Department of Obstetrics and Gynecology at Upper India Sugar Exchange Maternity Hospital, GSVM Medical College, Kanpur, after obtaining approval from the Ethics Committee (For Biomedical Health & Research), GSVM Medical College, Kanpur (Approval No. EC/403/Sept./2024). Participant recruitment was carried out from October 2024 to October 2025, and all enrolled women were followed prospectively until delivery. Written informed consent was obtained from all participants prior to enrollment.

The sample size was calculated using Cochran’s formula, considering an estimated prevalence of preeclampsia of 11%, with a 95% confidence interval and a 5% margin of error for the Kanpur population. The sample size was calculated to estimate the prevalence of preeclampsia and to prospectively evaluate the relationship between first-trimester markers and subsequent development of the disease. The calculated sample size was 151; however, 150 eligible pregnant women were included in the final analysis. Pregnant women with singleton pregnancies between 11 and 14 weeks of gestation attending routine first-trimester antenatal visits were enrolled consecutively. Gestational age was confirmed by crown-rump length measurement on first-trimester ultrasonography. Women with chronic hypertension, diabetes mellitus, renal, hepatic, or cardiovascular disorders, collagen vascular disease, documented platelet dysfunction, pregnancies conceived through in vitro fertilization, fetal chromosomal or structural anomalies, and women who developed other significant medical disorders during follow-up were excluded from the study.

Preeclampsia was defined as new-onset hypertension after 20 weeks of gestation, with or without proteinuria or maternal organ dysfunction [1]. At enrollment, demographic details, obstetric history, and relevant medical history were recorded, followed by general and systemic examination. Blood pressure was measured using a sphygmomanometer while the participant was seated comfortably after adequate rest, with the arm supported at heart level. Systolic and diastolic blood pressures were recorded, and MAP was calculated using the formula: MAP = DBP + 1/3 (SBP − DBP).

Transabdominal ultrasonography was performed using a Samsung RS 80A ultrasound system (Samsung Madison Co., Ltd, Seoul, Korea) equipped with a CA1-7A transducer. Uterine artery Doppler assessment was performed bilaterally at the level where the uterine artery crossed the external iliac artery [12]. Doppler waveforms were obtained from both uterine arteries, and the mean uterine artery PI was recorded.

Venous blood samples were collected at recruitment for the estimation of MPV and PAPP-A. MPV was analyzed using an automated hematology analyzer (Medonic M series). Serum PAPP-A estimation was carried out using chemiluminescent immunoassay methodology, and values were expressed both as absolute concentrations and multiples of the median (MoM) adjusted for gestational age.

All participants were followed through routine antenatal care until delivery. The primary outcome of the study was the development of preeclampsia during pregnancy.

Statistical analysis was performed using IBM SPSS Statistics for Windows, Version 27 (Released 2019; IBM Corp., Armonk, New York, United States). Continuous variables were expressed as mean ± standard deviation, while categorical variables were presented as frequencies and percentages. An independent samples t-test was used for comparison of continuous variables, whereas chi-square test or Fisher’s exact test was used for categorical variables. Pearson correlation analysis was performed to assess the association between predictive markers and preeclampsia. Binary logistic regression analysis was used to identify independent predictors of preeclampsia. Receiver operating characteristic (ROC) curve analysis and area under the curve (AUC) analysis were performed to evaluate the predictive performance of individual markers and combined models. A combined predictive model was constructed using multivariable logistic regression incorporating MAP, MPV, PAPP-A, and uterine artery Doppler PI, and predicted probabilities derived from this model were used for ROC analysis.

## Results

A total of 150 women were included in the study, of whom 131 were normotensive, and 19 had preeclampsia (Table 1). Most participants in both groups were aged between 26 and 35 years. In the normotensive group, 26.0% were aged 19-25 years, 72.5% were 26-35 years, and 1.5% were 36-43 years. Among women with preeclampsia, 10.5% were in the 19-25 age group, 63.2% were aged 26-35 years, and 26.3% were aged 36-43 years. Overall, 24.0% of participants were aged 19-25 years, 71.3% were aged 26-35 years, and 4.7% were aged 36-43 years. Based on BMI classification, in the normotensive group, 4.6% were underweight, 26.7% had normal BMI, 42.0% were overweight, and 26.7% were obese. In the preeclampsia group, 5.3% were underweight, 26.3% had normal BMI, 26.3% were overweight, and 42.1% were obese. Overall, 4.7% of participants were underweight, 26.7% had normal BMI, 40.0% were overweight, and 28.7% were obese. With respect to parity, 37.4% of normotensive women were primigravida and 62.6% were multigravida. Among women with preeclampsia, 21.1% were primigravida and 78.9% were multigravida. Overall, 35.3% of participants were primigravida, while 64.7% were multigravida. Regarding abortion history, 27.5% of normotensive women reported a history of abortion, whereas 72.5% did not. In the preeclampsia group, 36.8% had a history of abortion, and 63.2% did not. Overall, 28.7% of participants had a history of abortion, while 71.3% had none.

**Table 1:** Comparison of Baseline Maternal Characteristics between Normotensive and Preeclampsia Groups Fisher’s exact test was applied for age group due to low expected cell counts. The chi-square test was used for BMI, parity, and abortion history. BMI: body mass index

Variable	Category	Normotensive group (n = 131) n (%)	Preeclampsia (n = 19) n (%)	Total (n = 150) n (%)	Test Statistic	p-value
Age Group (years)	19–25	34 (26.0%)	2 (10.5%)	36 (24.0%)	Fisher’s exact test	0.011
	26–35	95 (72.5%)	12 (63.2%)	107 (71.3%)		
	36–43	2 (1.5%)	5 (26.3%)	7 (4.7%)		
	Total	131 (100)	19 (100)	150 (100)		
BMI (kg/m²)	Underweight	6 (4.6%)	1 (5.3%)	7 (4.7%)	χ² = 2.41	0.072
	Normal	35 (26.7%)	5 (26.3%)	40 (26.7%)		
	Overweight	55 (42.0%)	5 (26.3%)	60 (40.0%)		
	Obese	35 (26.7%)	8 (42.1%)	43 (28.7%)		
	Total	131 (100)	19 (100)	150 (100)		
Parity	Primi	49 (37.4%)	4 (21.1%)	53 (35.3%)	χ² = 1.29	0.256
	Multigravida	82 (62.6%)	15 (78.9%)	97 (64.7%)		
	Total	131 (100)	19 (100)	150 (100)		
Abortion History	Yes	36 (27.5%)	7 (36.8%)	43 (28.7%)	χ² = 0.33	0.567
	No	95 (72.5%)	12 (63.2%)	107 (71.3%)		
	Total	131 (100)	19 (100)	150 (100)		

The mean age of participants was 27.89 ± 4.53 years in the normotensive group and 30.21 ± 4.38 years in the preeclampsia group. The mean weight was 60.76 ± 11.49 kg among normotensive women and 64.00 ± 10.39 kg among those with preeclampsia. The mean BMI was 27.41 ± 5.63 kg/m² in the normotensive group and 28.72 ± 4.61 kg/m² in the preeclampsia group.

The comparison of clinical, biochemical, and Doppler parameters between normotensive and preeclampsia groups is shown in Table 2. The MAP was found to be higher in the preeclampsia group (103.31 ± 12.71 mmHg) than in the normotensive group (87.47 ± 10.45 mmHg); the difference is statistically significant (p< 0.001). The MPV was also higher in the preeclampsia group (12.00 ± 1.09 fL) than in the normotensive group (11.11 ± 1.61 fL); the difference is statistically significant (p = 0.004). The levels of PAPP-A were higher in the preeclampsia group (4.03 ± 3.26 mIU) than in the normotensive group (3.25 ± 2.97 mIU); the difference is statistically significant (p = 0.029). However, when adjusted to MoM, there is no significant difference between the two groups (p = 0.237). Regarding uterine artery Doppler, more cases of preeclampsia were found to be in the 95th percentile (36.8%) than in the normotensive group (22.9%); the distribution is statistically significant (p = 0.015). The preterm births were more in the preeclampsia group (42.1%) than in the normotensive group (28.2%); however, the difference is not statistically significant (p = 0.214).

**Table 2:** Comparison and Association of Clinical, Biochemical, and Doppler Parameters with Preeclampsia An independent samples t-test was used for continuous variables. Fisher’s exact test was applied for uterine artery Doppler PI due to low expected cell counts. The chi-square test was used for preterm delivery. MAP: mean arterial pressure; MPV: mean platelet volume; PAPP-A: pregnancy-associated plasma protein-A; PI: pulsatility index; SD: standard deviation.

Variable	Category/Group	Normotensive (n = 131)	Preeclampsia (n = 19)	Test Statistic	p-value
MAP (mmHg)	Mean ± SD	87.47 ± 10.45	103.31 ± 12.71	t = 6.01	<0.001
MPV (fL)	Mean ± SD	11.11 ± 1.61	12.00 ± 1.09	t = 2.92	0.004
PAPP-A (mIU)	Mean ± SD	3.25 ± 2.97	4.03 ± 3.26	t = 2.21	0.029
PAPP-A MoM	Mean ± SD	1.25 ± 1.12	1.58 ± 1.19	t = 1.19	0.237
Uterine Artery Doppler PI	5th Percentile	68 (51.9%)	10 (52.6%)	Fisher’s exact test	0.015
	50th Percentile	33 (25.2%)	2 (10.5%)		
	95th Percentile	30 (22.9%)	7 (36.8%)		
Preterm Delivery	Yes	37 (28.2%)	8 (42.1%)	χ² = 1.54	0.214
	No	94 (71.8%)	11 (57.9%)		

The correlation of predictive markers with preeclampsia is shown in Table 3. The relationship between MAP and preeclampsia was moderate, positive, and statistically significant (p < 0.001), with a correlation coefficient of 0.443. The relationship between MPV and preeclampsia was weak, positive, and statistically significant (p = 0.021), with a correlation coefficient of 0.188. The relationship between PAPP-A (MoM) and preeclampsia was weak, positive, but not statistically significant (p = 0.237), with a correlation coefficient of 0.097. The relationship between uterine artery Doppler PI and preeclampsia was weak but not statistically significant (p = 0.083), with a correlation coefficient of 0.143.

**Table 3:** Correlation of Predictive Markers with Preeclampsia MAP: mean arterial pressure; MPV: mean platelet volume; PAPP-A: pregnancy-associated plasma protein-A; MoM: multiples of the median; PI: pulsatility index.

Variable	Pearson Correlation (r)	p-value
MAP	0.443	<0.001
MPV	0.188	0.021
PAPP-A (MoM)	0.097	0.237
Uterine Artery Doppler PI	0.143	0.083

Binary logistic regression analysis for predictors of preeclampsia is shown in Table 4. Among the predictors that were assessed for their association with preeclampsia, the MPV was found to be significantly related to preeclampsia (p = 0.025) with an OR of 1.46 (95% CI: 1.049-2.031). PAPP-A was not found to be a significant independent predictor of preeclampsia on multivariate analysis. MAP, uterine artery Doppler PI, and BMI were not significantly related to preeclampsia (p > 0.05 for all three predictors).

**Table 4:** Binary Logistic Regression Analysis for Predictors of Preeclampsia OR: odds ratio; CI: confidence interval; MAP: mean arterial pressure; PI: pulsatility index; MPV: mean platelet volume; BMI: body mass index; PAPP-A: pregnancy-associated plasma protein-A.

Predictor	B	p-value	OR (Exp (B))	95% CI
PAPP-A	–0.016	0.381	0.98	0.52–1.87
MAP	0.003	0.857	1	0.97–1.03
Doppler PI	0.371	0.366	1.45	0.65–3.24
MPV	0.378	0.025	1.46	1.049–2.031
BMI	–0.007	0.973	0.99	0.67–1.48

The diagnostic performance and ROC curve analysis of the predictive markers and combined model are shown in Table 5. MAP demonstrated good predictive performance at a cut-off value of 96.7 mmHg, with a sensitivity of 78.9% and specificity of 81.7%. MPV showed the highest sensitivity (100%) at a cut-off value of 10.60 fL, although specificity was comparatively lower (38.2%). PAPP-A (MoM) demonstrated moderate predictive performance, with sensitivity and specificity values of 68.4% and 51.1%, respectively. Uterine artery Doppler PI showed a sensitivity of 88.9% and specificity of 31.5%. Among all models, the combined model incorporating MAP, MPV, PAPP-A, and uterine artery Doppler PI demonstrated the highest overall diagnostic accuracy, with a sensitivity of 83.3% and specificity of 81.5%.

**Table 5:** Diagnostic Performance of Predictive Markers for Prediction of Preeclampsia Cut-off values were determined using receiver operating characteristic (ROC) curve analysis based on optimal sensitivity and specificity. The positive predictive value (PPV) and negative predictive value (NPV) were calculated from the diagnostic performance analysis. The combined model represents multivariable logistic regression incorporating MAP, MPV, PAPP-A, and uterine artery Doppler PI. PPV: positive predictive value; NPV: negative predictive value; MAP: mean arterial pressure; MPV: mean platelet volume; PAPP-A: pregnancy-associated plasma protein-A; PI: pulsatility index; CI: confidence interval.

Marker	Cut-off Value	Sensitivity % (95% CI)	Specificity % (95% CI)	PPV % (95% CI)	NPV % (95% CI)
MAP	96.7 mmHg	78.9 (54.4–93.9)	81.7 (73.9–87.9)	38.5 (22.9–57.5)	96.4 (90.8–98.7)
MPV	10.60 fL	100 (82.4–100)	38.2 (29.9–47.2)	19.0 (11.5–29.8)	100 (92.8–100)
PAPP-A (MoM)	0.97	68.4 (43.4–87.4)	51.1 (42.2–59.9)	16.9 (9.5–28.2)	91.8 (82.8–96.9)
Uterine Artery Doppler PI	0.82	88.9 (65.3–98.6)	31.5 (23.6–40.5)	15.2 (8.9–24.8)	95.3 (84.5–98.8)
Combined Model	0.142	83.3 (58.6–96.4)	81.5 (73.6–87.7)	38.5 (23.4–56.5)	97.2 (91.4–99.1)

**Figure 1:**
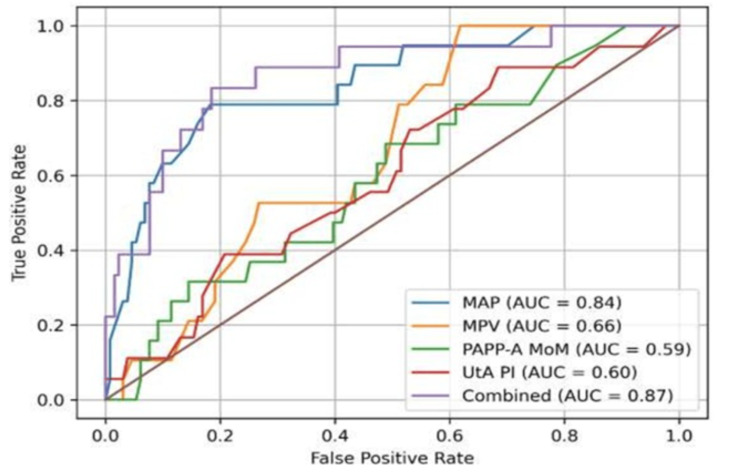
Receiver Operating Characteristic (ROC) Curves for Individual Predictive Markers and Combined Model in Predicting Preeclampsia ROC curves for individual predictive markers and the combined multivariable model incorporating mean arterial pressure (MAP), mean platelet volume (MPV), pregnancy-associated plasma protein-A (PAPP-A), and uterine artery Doppler pulsatility index (PI) for the prediction of preeclampsia. AUC: area under the curve

The AUC and statistical significance of individual and combined predictive markers for preeclampsia are shown in Table 6 and Figure 1. Among individual markers, MAP was found to have the highest predictive potential (AUC = 0.84, p < 0.001), followed by MPV (AUC = 0.66, p = 0.004). PAPP-A (MoM) was found to have lower predictive potential (AUC = 0.60, p = 0.029), while uterine artery Doppler PI had the lowest predictive potential (AUC = 0.59, p = 0.015). In contrast, combination models had better predictive potential compared to individual markers. All individual combinations of MAP, MPV, PAPP-A, and uterine artery Doppler PI had statistically significant predictive potential. Among them, a combination of MAP, MPV, PAPP-A, and uterine artery Doppler PI had the highest predictive potential (AUC = 0.87, p <0.001), followed by other individual combinations.

**Table 6:** AUC and Statistical Significance of Individual and Combined Predictive Markers for Preeclampsia AUC: area under the curve; CI: confidence interval; MAP: mean arterial pressure; MPV: mean platelet volume; PAPP-A: pregnancy-associated plasma protein-A; MoM: multiples of the median; UtA PI: uterine artery pulsatility index.

Marker/Combination	AUC	95% CI	p-value	Interpretation
MAP	0.84	0.75–0.93	<0.001	Good predictor
MPV	0.66	0.53–0.79	0.004	Moderate predictor
PAPP-A (MoM)	0.6	0.47–0.73	0.029	Weak predictor
Uterine Artery Doppler PI	0.59	0.46–0.72	0.015	Poor predictor
MAP + MPV	0.71	0.59–0.83	0.021	Significant
MAP + PAPP-A	0.74	0.62–0.85	0.008	Significant
MAP + UtA PI	0.72	0.60–0.84	0.013	Significant
MAP + MPV + PAPP-A	0.76	0.65–0.87	0.004	Significant
MAP + MPV + UtA PI	0.75	0.63–0.86	0.006	Significant
MAP + PAPP-A + UtA PI	0.77	0.66–0.88	0.003	Significant
MAP + MPV + PAPP-A + UtA PI	0.87	0.79–0.95	<0.001	Highest predictive performance

## Discussion

In this prospective study, early pregnancy biophysical, biochemical, and hematological parameters were evaluated for early prediction of preeclampsia. Of individual markers, MAP was found to be associated with future preeclampsia most strongly and had the best discriminative capacity on ROC analysis. The difference between cases and controls in first-trimester uterine artery Doppler PI, MPV, and serum PAPP-A levels was significant; PAPP-A was not significant when results were expressed in multiples of the median. MPV also emerged as an independent predictor on multivariate analysis. Notably, the combination of markers led to enhanced predictive performance with the highest detection rates with the multiparametric model (MAP, MPV, PAPP-A, and uterine artery Doppler PI).

Baseline characteristics

Maternal age was significantly higher among women who developed preeclampsia, with a greater proportion of cases observed in the higher age group. This implies that the risk of preeclampsia could be linked to the advancement of maternal age. Saxena et al. reported similar findings and found that most preeclampsia cases were identified in women aged 21-30 years, and Bairwa et al. reported that most of the preeclampsia cases were identified in women aged 25-30 years [13,14].

Other baseline variables such as weight, BMI, parity, and abortion history were similar in the preeclampsia and normotensive groups, implying that they did not significantly impact the development of preeclampsia in the current study.

Mean arterial pressure

MAP at an early stage of pregnancy in the current study was most closely related to the future development of preeclampsia, where the values were significantly higher in affected women. The correlation of MAP with preeclampsia and the highest level of discriminative ability on the ROC analysis also suggested that it is a good early predictor. Similar findings have been reported by Mayrink et al., who observed elevated early pregnancy MAP in women who later developed hypertensive disorders [15].

This similar predictive performance is not new; Demers et al. reported AUC values of 0.73 to 0.80 with first-trimester MAP [16]. The higher AUC observed in the present study may reflect the variation of the study population and clinical conditions of measurement. Moreover, the Fetal Medicine Foundation screening model uses MAP as an important biophysical parameter, which is emphasized to play a role in the early stages of pregnancies with placental dysfunction [17].

In spite of high discriminative capacity and excellent univariate association, MAP was not an independent predictor in multivariate analysis. This apparent discrepancy corresponds to the variance between univariate predictive performance and multivariate independence, in which shared variance with other markers would cause its own contribution to be attenuated in a combined model. Therefore, whereas MAP exhibited the best standalone predictive performance of individual markers, it is no longer a statistically significant predictor of preeclampsia when correcting for other variables and is best interpreted as a key component of a multiparametric screening approach rather than an isolated predictor.

Mean platelet volume

In the current study, the MPV was significantly higher among women who developed preeclampsia and demonstrated a positive relationship with the disease occurrence, which suggests that it is related to the early pathological changes. Though the MPV had a moderate discriminative ability on ROC analysis, it appeared to be an independent predictor of preeclampsia in the multivariate model, indicating that it has more predictive information than other clinical and biochemical markers. The observed 100 percent sensitivity needs to be assessed with care since the sample of preeclampsia cases is so small that it may not reflect the stability of this estimate and its generalizability.

It is possible that the increased MPV observed is due to platelet activation and endothelial dysfunction, which are important characteristics of preeclampsia pathogenesis. The results are similar, and Akhila et al. have found progressive MPV increases across gestation in women with hypertensive disorders, which suggests that platelet activation might change over the course of the disease [18]. Also, a meta-analysis study by Bellos et al. found considerably increased MPV in preeclamptic women with more severe cases, but the variability was found to be dependent on the severity of the disease and the time of measurement [19].

Although statistically significant, MPV did not predict as well as the other markers, even when used alone, because of its medium AUC. These results imply that though MPV might not be adequate and effective as a diagnostic tool, it has independent and clinically relevant information when used in multiparametric models to predict preeclampsia at an early stage.

PAPP-A as a predictor

The level of serum PAPP-A was higher in women who developed preeclampsia in the current study, but the difference in serum levels was not statistically significant when the level was expressed as MoM and had no significant correlation with preeclampsia. Besides this, PAPP-A has low discriminative power in ROC analysis, which implies that its standalone predictive performance in early pregnancy is modest.

On the other hand, earlier studies have indicated lower levels of PAPP-A associated with hypertensive pregnancy disorders during the first trimester. Meloni et al. found low PAPP-A levels predicted preeclampsia, and Nasrin et al. reported a better predictive performance when the PAPP-A values were in the lower range [20,21]. The seeming conflict in the direction of association can be attributed to variations in the way PAPP-A values are expressed and standardized. The majority of the past research findings indicate that PAPP-A was expressed as MoM, which controls the variations in gestational age and maternal traits, but the current research was mostly done with absolute concentrations. This variation in standardization may affect the direction and strength of the association that is observed. Moreover, PAPP-A is a dynamic biomarker that indicates the functionality of the placenta, and the concentrations can change based on the time when the samples are taken and the nature of the underlying population. The significance of standardized measurements of biomarkers and interpretation based on MoM in first-trimester screening models has been highlighted by Chaemsaithong et al. and could explain the differences in results across trials [17].

The elevated PAPP-A levels observed in the present study may reflect differences in placental and maternal adaptive responses rather than the classical mechanism of impaired trophoblastic invasion. This finding may be particularly relevant as most cases in our cohort likely represented term-onset disease, whereas reduced PAPP-A levels have been more consistently linked with early-onset preeclampsia. Similar observations have been reported by Uriel et al., suggesting that higher PAPP-A levels may be associated with distinct pathophysiological subtypes of hypertensive disorders in pregnancy [22]. Variability across studies may therefore be influenced by differences in disease phenotype, including early- versus late-onset presentation, as well as methodological factors such as assay variability and MoM standardization. Larger, well-characterized studies are needed to better understand this relationship and to clarify the role of PAPP-A in first-trimester screening.

Despite its limited standalone performance, PAPP-A remained part of the combined predictive model, suggesting a contributory rather than independent predictive role. The negative regression coefficient observed in the multivariate model, despite higher mean values in preeclampsia cases, may reflect adjustment for correlated variables and interactions between predictors rather than a true inverse relationship.

Uterine artery Doppler

In the current study, the uterine artery Doppler PI during the first trimester showed significant differences between the normotensive and preeclampsia groups, with a greater proportion of cases observed in the higher percentile category. This finding reflects increased uteroplacental vascular resistance and supports the role of impaired placentation in the pathogenesis of hypertensive disorders of pregnancy.

Past research has demonstrated that abnormal uterine artery Doppler measurements during the early pregnancy are associated with the future development of preeclampsia. According to Khong et al., first-trimester Doppler showed moderate predictive performance, particularly in low-risk populations [23]. On the same note, Shivrayan et al. and Kushtagi et al. also found that there was a higher risk of hypertensive disorders with a higher Doppler resistance index [24,25].

Nevertheless, in the current study, uterine artery Doppler PI failed to show significant correlation and was not a significant independent predictor in the multivariate analysis, which implies that it has a limited standalone predictive utility. Also, it had low discriminative ability in ROC analysis compared to other markers. These data indicate that Doppler PI is not an adequate screening tool in itself, though it indicates underlying dysfunction of the placenta.

Doppler of the uterine artery has been known to predict performance better in combination with other markers. The study by Poon et al. showed that the use of Doppler indices with maternal and biochemical parameters was more likely to detect preeclampsia, whereas Yucel et al. also found a higher predictive accuracy with the combined models [8,26].

Combined model

The four-marker model incorporating MAP, MPV, PAPP-A, and uterine artery Doppler PI demonstrated the highest predictive performance within the study population, with better sensitivity and specificity than the single markers. This observation underscores the complexity of preeclampsia, in which combinations of biophysical, biochemical, and hematological measures enhance early risk stratification.

Although each of the markers was diverse in predictive capability, their combination resulted in a clear improvement in overall diagnostic accuracy, implying that these parameters represent complementary pathophysiological mechanisms (maternal hemodynamic alterations, placental dysfunction, and endothelial activation).

These results are in line with other studies. Poon et al. showed that combined screening approaches integrating maternal characteristics, uterine artery Doppler, and biochemical markers significantly improve detection rates for preeclampsia [8]. Equally, Kumar et al. found an increase in predictive accuracy in the case of multiparametric models [27]. Moreover, Chaemsaithong et al. emphasized that first-trimester prediction models using multiple parameters, including the Fetal Medicine Foundation model, have a significantly higher detection rate than the risk factor-based screening strategies [17].

Overall, the available findings of the current research support the application of combined first-trimester screening models to identify women at risk of Preeclampsia in the early phases, instead of using individual parameters.

If validated in larger populations, such screening approaches may assist in identifying women who could benefit from preventive strategies such as low-dose aspirin prophylaxis before 16 weeks of gestation. This highlights the role of multiparametric screening not only in prediction but also in guiding preventive strategies [11].

The current research has certain limitations. The sample size was determined based on the estimated prevalence of preeclampsia, and only 19 participants developed the condition during follow-up. Although the combined model demonstrated encouraging predictive performance, the relatively small number of outcome events may limit the generalizability of the findings and warrant cautious interpretation of the multivariable analyses. The study is a single-center one, and therefore, the findings might be affected by the population-specific characteristics, which creates a possible selection bias. Moreover, the inconsistencies in the biomarker measurements and the absence of external validation can also affect the repeatability and the broader use of the predictive model. Although these are the limitations of this study, one of the strengths is the prospective nature and thorough assessment of multiple predictive markers at the first trimester, which demonstrates the clinical utility of a multiparametric approach in early detection of women at risk of preeclampsia.

## Conclusions

Early pregnancy evaluation of biophysical, biochemical, and hematological parameters offers a practical approach for predicting preeclampsia. MAP demonstrated the highest discriminative ability among individual markers, while MPV and PAPP-A contributed additional predictive value despite limited standalone performance. Uterine artery Doppler findings reflected underlying placental dysfunction but showed relatively modest independent utility.

The combined use of these parameters in a multiparametric model demonstrated superior predictive accuracy compared to individual markers. These findings suggest that first-trimester combined screening approaches may improve early risk stratification for preeclampsia, particularly in settings with a high disease burden. However, larger prospective studies with external validation are required before broader clinical implementation can be recommended.
